# Influence of circadian rhythm on effects induced by mechanical strain in periodontal ligament cells

**DOI:** 10.1007/s00056-024-00542-1

**Published:** 2024-08-12

**Authors:** Lena I. Peters, Jana Marciniak, Eric Kutschera, Caio Luiz, Erika Calvano Küchler, Christian Kirschneck, Andreas Jäger, Svenja Beisel-Memmert

**Affiliations:** 1 Department of Orthodontics, Center of Dento-Maxillo-Facial Medicine, University Hospital Bonn, Medical Faculty, Welschnonnenstr. 17, 53111 Bonn, Germany; 2 Department of Pediatric Dentistry, School of Dentistry of Ribeirão Preto, Ribeirão Preto, Brazil; 3 School of Dentistry, Universidade Tuiuti do Paraná, Curitiba, Brazil

**Keywords:** Tooth movement, PDL-cells, Mechanical stimulation, Clock genes, Circadian rhythm, Zahnbewegung, PDL-Zellen, Mechanische Stimulation, Clock-Gene, Zirkadiane Rhythmik

## Abstract

**Purpose:**

The aim of this study was to investigate the influence of mechanical strain on clock gene function in periodontal ligament (PDL) cells. Furthermore, we wanted to analyze whether effects induced by mechanical stress vary in relation to the circadian rhythm.

**Methods:**

Human PDL fibroblasts were synchronized in their circadian rhythm with dexamethasone and stretched over 24 h. Unstretched cells served as controls. Gene expression of the core clock genes were analyzed at 4 h intervals by quantitative real-time polymerase chain reaction (qRT-PCR). Time points 0 h (group SI1) and 12 h (group SI2) after synchronization served as starting points of a 4 h force application period. Collagen-1α (COL-1α/Col-1α), interleukin-1β (IL1-β), and runt-related transcription factor 2 (RUNX2/Runx2) were assessed by qRT-PCR and enzyme-linked immunosorbent assay (ELISA) after 2 and 4 h. Statistical analysis comprised one-way analysis of variance (ANOVA) and post hoc tests.

**Results:**

After synchronization, the typical pattern for clock genes was visible in control cells over the 24 h period. This pattern was significantly altered by mechanical strain. Under tensile stress, ARNTL gene expression was reduced, while Per1 and 2 gene expression were upregulated. In addition, mechanical stress had a differential effect on the expression of Col-1α and IL1‑β depending on its initiation within the circadian rhythm (group SI1 vs group SI2). For RUNX2, no significant differences in the two groups were observed.

**Conclusion:**

Our results suggest that mechanical stress affects the molecular peripheral oscillator of PDL cells. Vice versa, the circadian rhythm also seems to partially influence the effects that mechanical stress exerts on PDL cells.

## Abstract (de)

**Ziel:**

Ziel dieser Arbeit war es, den Einfluss von mechanischer Belastung, wie sie auch im Rahmen einer kieferorthopädischen Zahnbewegung auf das Parodontalligament (PDL) appliziert wird, auf Funktionen von Clock-Genen zu untersuchen. Zusätzlich sollte analysiert werden, ob sich eine mechanische Belastung in Abhängigkeit der zirkadianen Rhythmik unterschiedlich auf wichtige Proteine der PDL-Zellen auswirkt.

**Material und Methoden:**

Die periphere zirkadiane Rhythmik humaner PDL-Zellen wurde mittels Dexamethason synchronisiert und einer statischen Dehnung von 20% über bis zu 24 h ausgesetzt. Parallel wurde eine Kontrollgruppe ohne Dehnung angesetzt. In Zeitintervallen von 4 h wurde die Genexpression der Clock-Gene mittels qRT-PCR („quantitative real-time polymerase chain reaction“) bestimmt. Weiter wurden die Zellen einem Dehnungsintervall von 4 h zu den Zeitpunkten 0 h (Gruppe SI1) und 12 h (Gruppe SI2) nach Synchronisation ausgesetzt. Die Expression der Gene Collagen-1α (Col-1α), Interleukin-1β (IL1-β) und Runt-verwandter Transkriptionsfaktor 2 (RUNX2) wurde jeweils nach 2 und 4 h mittels qRT-PCR und ELISA („enzyme-linked immunosorbent assay“) quantifiziert. Die statistische Analyse erfolgte über eine einseitige Varianzanalyse (ANOVA) und Post-hoc-Tests.

**Ergebnisse:**

In den Kontrollzellen war nach Synchronisation das für die Clock-Gene typische Muster im Verlauf der 24 h erkennbar. Dieses wurde durch die mechanische Belastung signifikant verändert. Unter Zugbelastung wurde eine Verringerung der ARNTL-Genexpression verzeichnet, während die Per1- und Per2-Genexpression hochreguliert wurden. Die mechanische Belastung hatte abhängig von der Initiierung nach Synchronisation (Gruppe SI1 vs. Gruppe SI2) einen unterschiedlichen Einfluss auf die Expression von Col-1α und IL1‑β. Für Runx2 wurde in beiden Gruppen kein Unterschied beobachtet.

**Schlussfolgerung:**

Die Ergebnisse legen nahe, dass mechanische Belastung den molekularen peripheren Oszillator der PDL-Zellen beeinflusst. Umgekehrt scheint auch die zirkadiane Rhythmik die Auswirkungen von mechanischem Stress auf PDL-Zellen teilweise zu beeinflussen.

## Introduction

Orthodontic tooth movement (OTM) is induced by forces exerted by orthodontic appliances. During OTM, remodeling processes take place in the periodontal ligament (PDL) and surrounding bone. The mechanical force applied to the tooth causes compressive, but also tensile strain in various areas of the PDL. This results in biomechanical stress on periodontal tissues and especially on cells in the PDL. These PDL cells play an important role in the control of resorption and apposition of bone induced by mechanical stress [41].

Mechanical stress also results in sterile inflammation that includes an associated release of inflammatory mediators such as cytokines including interleukin 1-beta (IL-1β) [13, 19, 43, 46]. The release of these messenger substances activates both osteoclasts and osteoblasts and causes bone remodeling [3, 16]. In addition, dependent on the amount of force applied, specific factors such as runt-related transcription factor (RUNX2/Runx2) or collagen-1α (COL-1α/Col-1α) are increased or inhibited [48].

Runx2 plays an important role in bone development. It is co-decisive for osteoblast differentiation [10, 64, 65] and it has been shown previously in vitro and in vivo that RUNX2 gene expression is increased by mechanical forces in PDL cells and osteoblasts, whereas reduced exposure to mechanical strain, i.e., microgravity, decreases Runx2 expression [8, 65].

One of the most important proteins of the extracellular matrix in the periodontium is Col-1α, predominantly produced by osteoblasts and PDL cells, which is responsible for the elastic properties of the periodontium [9]. Collagen is increasingly produced in the stretched parts of the periodontium especially during tooth movement, while collagen production was shown to be reduced on the pressure side [23].

Circadian rhythms define endogenous cycle processes that occur in a 24 h period. Environmental stimuli can influence circadian rhythms and are described as “Zeitgeber” [14]. In mammals, a crucial Zeitgeber is daylight, which influences the body’s central clock in the suprachiasmatic nucleus (SCN) in the front of the hypothalamus [40]. The SCN is seen as the main pacemaker of the human body, controlling other peripheral clocks in organs and tissues via neuronal and hormonal signaling pathways, a process called ‘entrainment’ [1, 11, 59]. Prerequisite for the maintenance of a circadian rhythm in peripheral tissues is the group of clock genes. They form the peripheral oscillator at the molecular level. Different clock genes are part of activating and inhibiting feedback loops. The main feedback system comprises the transcription factors aryl hydrocarbon receptor nuclear translocator-like protein 1 (ARNTL) or brain and muscle ARNT-like 1 (BMAL1), and circadian locomotor output cycles kaput (CLOCK). They induce the formation of the period (PER; PER1/PER2/PER3) and cryptochrome genes (CRY1/CRY2). These in turn form a heterodimer together and directly inhibit the complex of CLOCK and ARNTL. Thus, the activating loop is formed by ARNTL and CLOCK, while the inhibitory feedback loop is dependent on the PER and CRY complex [26, 30, 49, 61]. This feedback loop, which is “straightforward” at the molecular level, is also described as the “core” oscillator mechanism in mammals [30].

It was shown that some of the clock genes are also involved in various other important cell functions [14]. Thus, the circadian rhythm is a crucial factor for physiological and pathophysiological processes in the human body and has an impact on diseases such as diabetes, cardiovascular disorders, sleep disorders, and also cancer [15, 28, 42, 44, 47, 53, 54].

There is evidence that circadian rhythmicity is also of relevance in oral cell homeostasis. For example, dental pulp cells, ameloblasts, odontoblasts, cementoblasts, gingival fibroblasts, and PDL cells are influenced by the circadian rhythm [17, 20–22, 60, 62, 63]. It was also shown that the proliferation of alveolar bone and tooth cementum in mice have a circadian rhythm [51]. Thereby, the expression of development- and remodeling-related genes for these tissues like OPG, RANKL, RUNX2, OCN, OPN, and COL-1α present a circadian rhythm, all of which were proven to share a correlation with the fluctuation of the expression of different clock genes [29, 56].

That the expression of clock genes follows a circadian pattern after synchronization in PDL cells was already shown [17].

The current study focused on the investigation of a possible influence of mechanical stress on the clock genes in PDL cells. Furthermore, we aimed to analyze whether tensile strain has different effects on elementary regulatory factors of PDL remodeling (IL-1β, RUNX2, COL-1α) depending on the phase of the circadian rhythm. We hypothesized that a differential influence of mechanical stress on PDL homeostasis can be assumed depending on the circadian rhythm.

## Materials and methods

### Preparation and cultivation of periodontal ligament cells

A human PDL fibroblast cell line was acquired in the second passage from the company Lonza (Lonza Ltd., Basel, Switzerland; # CC-7049). Passage 6 cells were used and cultured on special cell culture plates with stretchable collagen membranes (Flexcell International, Hillsborough, NC, USA). Dulbecco minimal essential medium (DMEM, Invitrogen Karlsruhe, Germany) with the addition of 10% serum (FBS), 1% Pepstrep 100 µg/ml, Plasmocin (Invivogen, Toulouse, France), and vitamin C served as culture medium. The medium was changed every 3–4 days. Cells were cultivated in a 37 °C incubator with 5% CO_2_. One day before the start of the experiment, the FBS addition in DMEM was reduced to 1% FBS.

### Cell synchronization procedure

Two cell groups (control group and STSH group) were synchronized by an exposure to dexamethasone (100 nM Sigma-Aldrich Chemie GmbH, Munich, Germany) for 30 min at time point t0 [5, 17]. After the synchronization period, the medium was changed again to DMEM containing 1% FBS. Static mechanical strain was applied by a specially developed stretching apparatus developed at the University of Bonn [31] with a static stretch of 20%. Over a period of 24 h, fibroblasts were harvested at 4 h intervals.

To investigate whether a mechanical force applied at different time points after synchronization has different effects on regulatory factors, a 4 h stretching period was started at t0, directly after synchronization (stretching interval 1, group SI1) as well as at t12, 12 h after synchronization (stretching interval 2, group SI2). In both groups, cells were harvested after 2 and 4h.

### Analysis of gene expression

After the experiments, cells were harvested with RLT buffer and β‑mercaptoethanol and RNA isolation was performed with the RNeasy-mini Kit (RNeasy Protect-Minikits, Qiagen, Hilden, Germany). Hereafter, a nano-drop Spectrometer (Bio-Rad Laboratories, Munich, Germany) was used to measure RNA concentration at a wavelength of 260 nm.

Conversion to cDNA was carried out using the iScrpit kit (iScrpit cDNA synthesis Skript, Bio-Rad Laboratories, Munich, Germany). All samples were cooled on ice during RNA isolation and cDNA production. The manufacturer’s instructions were always followed when using the kits.

The following clock genes were analyzed: CLOCK1, ARNTL, PER1, and PER2 (# QT00054481, # QT00011844, # QT00069265, # QT00011207, Qiagen,Hilden, Germany). Clock gene detection was done with a quantitative real-time PCR (qRT-PCR) at each of the seven time points. The control gene was glyceraldehyde-3-phosphate dehydrogenase. For the qRT-PCR, 2.5 µl of QuantiTect Primer (QuantiTect Primer Assay, Qiagen, Hilden, Germany), 12.5 µl QuantiTect Syber Green Master-Mix (QuantiTect SYBR Green PCR Kit, Qiagen, Hilden, Germany), 5 µl nuclease-free water (Qiagen, Hilden, Germany), and 5 µl of the sample, respectively, were utilized in an iCycler IQ5 (Bio-Rad Laboratories, Hercules, CA, USA). The PCR protocol started with initial heating to 95 °C for 5 min for enzyme activation. The first 50 cycles included denaturation at 95 °C for 10 s and a combined primer hybridization and extension at 60 °C for 30 s per cycle (Qiagen QuantiTect protocol). A melting curve analysis was performed after each run. The iCycler IQ5 (Bio-Rad Laboratories, Hercules, CA, USA) was used for the qRT-PCR. Specific gene expression of the clock genes was calculated with the respective control using the comparative cycle threshold method [37].

Gene expression analysis of the genes (RUNX2, Col-1α, and IL-1β) was carried out analogously to that of the clock genes.

### Analysis of protein expression of the regulatory factors

Cell supernatant was collected and protein detection was performed. The following ELISA kits were used: RUNX2—RUNX2 Elisa Kit (human; Aviva Systems Biology, San Diego, CA, USA), Col-1α—Human Pro-Collagen I alpha DuoSet Elisa (R&D systems, Wiesbaden-Nordenstadt, Germany), IL-1β—Human IL-1β Elisa Kit (Invitrogen, Thermo Fischer Scientific, Vienna, Austria). The measurement was carried out with an ELISA reader (Epoch, Bio Tek Instruments, Winooski, VT, USA) at a wavelength of 450 nm. The reference wavelength was 620 nm. The manufacturer’s instructions were followed for each measurement.

### Statistics

For each experiment, triplets of each sample were used and replicated at least three times. Statistical analyses were performed using Graphpad Prism (version 7.00 for Windows, GraphPad Software, San Diego, CA, USA). Data are presented as means with standard error of the mean (SEM). Multiple comparisons were carried out using Dunnett’s test. In addition, a one-way analysis of variance (ANOVA) was performed, followed by a post hoc Sidak test. The level of significance was set at *p* < 0.05.

## Results

### Regulation of the molecular peripheral oscillator

**Fig. 1:**
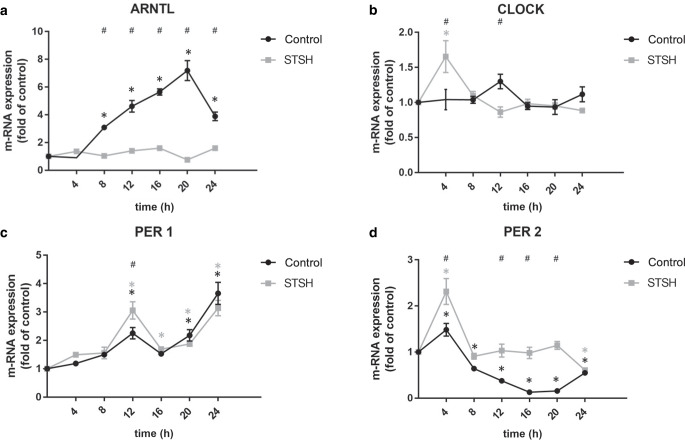
Longitudinal data for clock gene expression of ARNTL (**a**), CLOCK (**b**), PER1 (**c**), and PER2 (**d**) of stretched groups (STSH) compared to unstretched controls over 24 h after synchronization with dexamethasone. *Rhomb* statistically significant differences (*p* < 0.05) between stretched and unstretched group according to Sidak test, *Asterisk* statistically significant difference (*p* < 0.05) over time compared to t0 according to Dunnett’s test Longitudinale Darstellung der Clock-Genexpression von ARNTL (**a**), CLOCK (**b**), PER1 (**c**) und PER2 (**d**) von gedehnten PDL-Zellen (STSH) im Vergleich zur ungedehnten Kontrollgruppe. Die Dehnungsexposition erfolgte über 24 h nach Synchronisation mit Dexamethason. *Raute* Statistisch signifikanter Unterschied (*p* < 0,05) zwischen der Kontrollgruppe und der gedehnten Gruppe (Sidak-Test). *Asterisk* Statistisch signifikante Unterschiede (*p* < 0,05) über die Zeit verglichen mit t0 (Dunnett-Test)

In the control cells, significant changes in the expression of the clock genes ARNTL, PER1, and PER2 were observed over the course of 24 h after synchronization (Fig. 1a, c, d). While ARNTL was significantly increased at each measured time point from 8 h (*p* < 0.0001) onwards, PER2 showed a significant inhibition at each measured time point from 12 h (*p* < 0.0002) onwards. At time point 4 h (*p* < 0.0055), there was a significant increase in the expression of PER2. Gene expression of PER1 was significantly enhanced at the 12 h (*p* < 0.0001), 20 h (*p* < 0.0001), and 24 h (*p* < 0.0001) time points. Interestingly, there was no significant change in CLOCK gene expression (Fig. 1b). Compared to the control cells, mechanical stimulation significantly affected the gene expression of all CLOCK genes analyzed. ARNTL gene expression was reduced by mechanical stimulation at every measured time point starting from 8 h (*p* < 0.0001) onwards. Gene expression of CLOCK was significantly increased by stretch at the 4 h time point (*p* < 0.0001) after synchronization and significantly decreased at 12 h (*p* < 0.01). Stretching significantly elevated gene expression of PER1 at time point 12 h (*p* < 0.017) after synchronization and of PER2 at time points 4 h (*p* < 0.0001), 12 h (*p* < 0.0001), 16 h (*p* < 0.0001), and 20 h (*p* < 0.0001).

### Expression changes in different phases of the circadian cycle

**Fig. 2:**
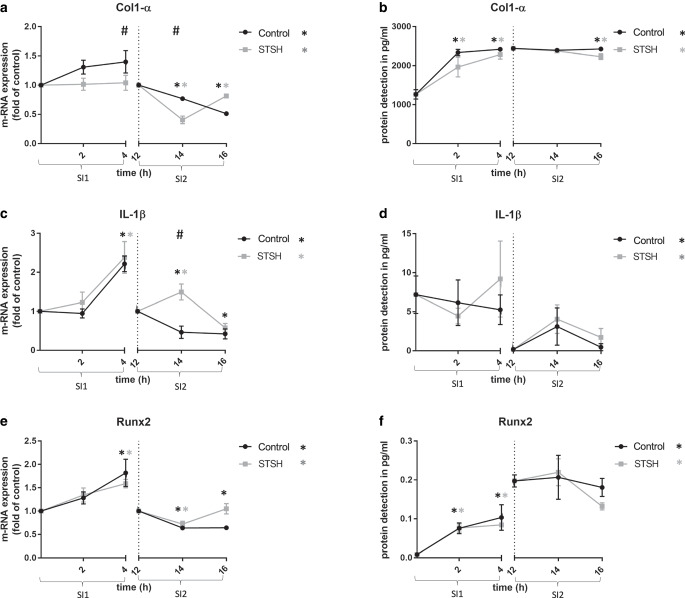
Longitudinal data for regulatory factor gene expression and protein detection for Col1-α (**a**, **b**), IL1-β (**c**, **d**), and Runx2 (**e**, **f**). Unstretched controls were compared with cells submitted to mechanical load starting at time points t0 (group SI1) and t12 (group SI2) after synchronization with dexamethasone. *Rhomb* statistically significant differences (*p* < 0.05) between stretched and unstretched group according to Sidak test, *Asterisk* statistically significant difference (*p* < 0.05) over time as compared to t0 or t12 according to Dunnett’s test Longitudinale Darstellung der Genexpression und Proteinnachweis mittels Elisa der regulatorischen Faktoren Col-1α (**a**, **b**), IL1-β (**c**,**d**) und Runx2 (**e**, **f**). Zellen der ungedehnten Kontrollgruppe wurden mit mechanisch stimulierten Zellen verglichen. Die Startpunkte der 2‑ und 4‑stündigen mechanischen Belastung waren jeweils an t0 (Gruppe S1) bzw. an t12 (Gruppe S2) nach Dexamethason-Synchronisation. *Raute* Statistisch signifikante Unterschiede (*p* < 0,05) zwischen der Kontrollgruppe und der gedehnten Gruppe (Sidak-Test), *Asterisk* statistisch signifikanter Unterschied (*p* < 0,05) über die Zeit verglichen mit t0 oder t12 (Dunnett-Test)

Based on the results of clock gene expression analysis in the first part of this study, two different starting points for mechanical loading after synchronization were chosen for the second part. These time points were t0 (group SI1) and t12 (group SI2) after synchronization. The time of mechanical stimulation was 4 h and measurement took place after 2 and 4 h (Fig. 2a–f).

Gene expression of COL-1α showed no significant change in group SI1 after 2 and 4 h Hs compared to t0. However, a trend If increased gene expression could be detected. In group SI2, after 2 and 4 h (*p* < 0.0181), a significant reduction in gene expression was evident as compared to t12 (Fig. 2a). At the protein level, an increased expression was significant in group SI1 after 2 h (*p* < 0.0001) and after 4 h (*p* < 0.0001) as compared to t0. In group SI2, a slight but significant increase (*p* < 0.0065) in protein expression was detectable after 4 h.

At the gene expression level, compared with the control cells, a significant reduction in COL-1α gene expression was detectable due to mechanical stress ingroup SI1 after 4 h (*p* < 0.0210) and in group SI2 after 2 h (*p* < 0.0181). At the protein level, no difference in Col-1α protein expression was detectable between the two groups exposed to mechanical strain and the control groups.

IL-1β gene expression was significantly upregulated after 4 h (*p* < 0.0002) in group SI1 (Fig. 2c). However, in group SI2, after 2 h (*p* < 0.01) as well as after 4 h (*p* < 0.02), a significant downregulation was observed in comparison to t12.

Stretching did not significantly change IL-1β gene expression in group SI1 as compared to the control. However, in group SI2, after 2 h (*p* < 0.0006) of stretching, a significant increase in gene expression was measured. At the protein level, the scattering within each group and experiment was too high, so that no significant change could be measured at any time point (Fig. 2d).

An elevated gene expression of RUNX2 was detected after 4 h (*p* < 0.0002) in group SI1, whereas expression of this gene was decreased in group SI2 after 2 h (*p* < 0.0007) and after 4 h (*p* < 0.0076; Fig. 2e). An increased protein level of Runx2 was also detected by the ELISA assay in group SI1 after 2 h (*p* < 0.0005) and after 4 h (*p* < 0.0005; Fig. 2f). However, protein levels were not significantly altered after 2 h or 4 h in group SI2 compared with t12.

## Discussion

In this study, the influence of mechanically induced stress on the main players of peripheral circadian rhythm of PDL fibroblasts was investigated. Static stretching altered gene expression of all clock genes investigated, suggesting an influence of mechanical stress on these peripheral messengers of the circadian rhythm. However, vice versa, the circadian rhythm also seemed to influence the effects that a mechanical force exerted on PDL cells. This was apparent by a differential regulation of three important regulatory factors, depending on the onset of mechanical strain in the course of the circadian rhythm.

Also, the molecular concept of a circadian rhythm in PDL fibroblasts could be observed and significant changes of the clock genes ARNTL, PER1, and PER2 over the course of 24 h after synchronization became evident. As already demonstrated by Hilbert et al., the peripheral circadian rhythm is an important process that is controlled in PDL fibroblasts by a cyclic regulation of the clock genes [17]. Also in this study, regulation occurred after synchronization of PDL fibroblasts with the help of dexamethasone. An interesting finding was that the gene expression of ARNTL and Per2 showed an opposite course. This opposite regulation is a feature of the peripheral molecular clock, as ARNTL is a so-called positive factor of the molecular feedback loop of the circadian clock, whereas the Period genes belong to the corresponding negative factors [50]. Also, in the paper of Hilbert et al., regulations of ARNTL and PER2 were found in particular, whereas CLOCK and PER1 were influenced to lesser extents. This could suggest that ARNTL and PER2 are profoundly important for regulation of the molecular clock in PDL fibroblasts, but this assumption should be further investigated, because the experiments of Hilbert et al. and the experiments of the present study were performed with the same PDL cells from Lonza in the same laboratory.

Mechanical strain increased the gene expression for the negative factors of the circadian clock PER1 and PER2, whereas the positive factor ARNTL was reduced by mechanical stimulation at every time point measured from the 8 h time point onward. CLOCK gene expression was also affected by mechanical stimulation. Furthermore, 4 h after synchronization, an increase was detected, whereas a reduction was observed after 12 h. The influence of mechanical stimulation on molecular clock genes which our results suggest are consistent with the existing literature. Qin et al. examined rat PDL cells after orthodontic tooth movement. In their in vivo study, an increase in the expression of PER1 was demonstrated to be induced by mechanical stimulation [38].

In addition to the question of whether a mechanical force affects the peripheral circadian clock, we tried to determine whether the circadian clock alters the effects that mechanical forces induce in PDL fibroblasts. Therefore, mechanical strain was initiated at different time points after synchronization and the effects of the mechanical force on key regulatory factors were examined. We picked a second time interval 12 h after synchronization for the stimulation, because ARNTL and PER2 had demonstrated an opposite regulation starting at this time point in the control cells. Furthermore, Hilbert et al. already showed that the time point 12 h after synchronization was well suited to monitor gene expression changes of regulatory factors in PDL cells in the course of the circadian rhythm [17].

Starting mechanical strain at t0 (directly following synchronization, group SI1) after 2 and 4 h, there was an increase in protein expression of Col-1α compared to t0, whereas the same mechanical stimulation starting at t12 induced a reduced gene expression (after 2 and 4 h) and protein expression (after 4 h) compared to t12. Hilbert et al. also discovered reduced gene expression of COL-1α 12 h after synchronization of the cells. These results provide evidence for an influence of the circadian rhythm on Col-1α synthesis. Qin et al. also investigated the regulation of Col-1α synthesis in relation to the circadian rhythm, but in a rat model. The authors found a reduction in COL-1α gene expression at each measured time point in the 12–18 h time interval, although this reduction did not reach significance. As mentioned in the introduction, Col-1α is critical in cell homeostasis and connective tissue turnover [12, 18, 33]. Interestingly, at the transcriptional level, the mechanical stress had an inhibitory effect on COL-1α gene expression independent of the time of its onset within the circadian cycle. On the other hand, at the protein level, an increase in protein expression was observed after mechanical stimulation in group SI2. This is in accordance with findings of another study which also showed that stretching of PDL cells increased the protein biosynthesis of Col-1α [18]. Qin et al. observed an upregulation of COL-1α gene expression after 3 and 21 h following mechanical load in vivo [38]. Of course, in that in vivo experiment, it was not possible to locally separate the true types of strain triggered by the force, being it tension or compression, as in our in vitro approach. Moreover, in their study continuous force was applied, while in our experimental setup, we picked the force onset at different time points after synchronization. Thus, our results are difficult to compare with those of Qin et al. [38].

Runx2 is a critical factor in osteoblast differentiation and it is important for bone remodeling during orthodontic tooth movement [64]. Our results showed an upregulation of RUNX2 gene expression 4 h after synchronization in group SI1, whereas in group SI2, gene expression was decreased after 2 h as well as after 4 h of mechanical strain. Again, parallels can be drawn to the results of Hilbert et al., as they found a decreased gene expression for RUNX2 12 h after synchronization compared to t0 [17]. Other studies also found Runx2, which is also a transcriptional factor crucial for the differentiation of ameloblasts, to be upregulated in dependence of other clock genes such as Nr1d1 [4, 52]. Mechanical strain, like tension, has repeatedly been shown to lead to increased gene expression of RUNX2 alongside cell proliferation. This has been demonstrated in murine stem cells and in human PDL cells [2, 48]. However, the magnitude of the tensile strain plays a crucial role. Sun et al. found that excessive elongation of the cell decreased the gene expression of RUNX2 [48]. We used a strain of the cells leading to an elongation of about 20%, which is considered to belong the higher spectrum of tissue strain induced by orthodontic forces [24]. It was noticeable that for Runx2 no significant differences, neither at the gene nor the protein level, resulted from mechanical stimulation. Hence, further studies with variable stretch intensities and time intervals are necessary to gain further insights into the effects of mechanical strain on RUNX2 in dependence of the circadian rhythm.

Along with other interleukins, IL1‑β is an important inflammatory marker detectable in the remodeling processes of mechanically induced tooth movement in the periodontium [13]. Similar to Hilbert et al., in our control group an increase in gene expression of IL1‑β was seen 2 and 4 h after synchronization and then a decrease in the time slot from t12 to t16a [17]. IL1‑β has already been linked to the circadian rhythm by other studies. It is an important factor in sleep rhythm and can interfere with spontaneous sleep, when inhibited [25]. Alterations of IL-1β during the course of the circadian rhythm have also been demonstrated in oral saliva [39]. The mechanical force triggered an increase in gene expression in group SI2 from starting point t12 onwards with a maximum value measured 2 h after mechanical strain. It is already known from several studies that intermittent forces promote IL-1β production, which would be consistent with our results [34, 35].

For comparable measurements, we followed the protocol of Hilbert et al. regarding the synchronization protocol [17]. Since our cell culture lacks the central Zeitgeber, we used an artificial pacemaker to induce synchronization of our cells. Balsalobre et al. showed that dexamethasone as a serum component causes high reproducibility of circadian synchronization [6, 7]. With dexamethasone stimulations during different time points in the course of a 24 h phase, a change in circadian rhythm has to be expected. This may favor but also disrupt the circadian rhythm [57]. Other stimuli such as Ca^2+^, protein kinase C, and glucocorticoid hormones are also able to induce fibroblast synchronization, but these are already components of the used serum [5, 6]. Additionally, the possible implications of the supplements in our DMEM have to be discussed. We added 1% serum, 1% Pepstrep 100 µg/ml, Plasmocin, and vitamin C. We know from previous studies that serum, for example, can influence the circadian rhythm [6]. In addition, central and peripheral oscillators can be influenced differently by factors such as nutrients and feeding cycles [36]. The other supplements, such as vitamin C as an antioxidant, are also likely to influence the circadian rhythm [55]. Detailed studies investigating the causal relationship between circadian rhythm and oxidative stress are still lacking. Therefore, we cannot exclude that the administration of different supplements in our paper and the work of Hilbert et al. influenced the circadian rhythm of PDL cells [17].

Furthermore, the use of the right cell passage is important, as cell senescence has an impact on circadian expression of clock genes in vitro [27]. Kunieda et al. reported an attenuated circadian expression of clock genes in senescent passages compared to young cell passages in cultured human aortic vascular smooth muscle cells. Shimizu et al. investigated cell senescence in human periodontal ligament cells during mechanical loading [45]. They characterized passages 5–6 as young passages. From passage 15 onwards, the cell doubling population slows down. Passages over 22 were identified as senescent passages. Therefore, in regard to cell senescence, there should not be attenuated effects on the clock gene expression in our experiments as we used passage 6, a comparable passage to Hilbert et al., who used passage 4 [17].

Additionally, there are also disadvantages of our chosen in vitro approach. The in vivo study performed by Qin et al. certainly offers a more comprehensive picture of the overall influences of the circadian rhythm on the periodontal tissues. However, especially for such a scarcely researched topic as the interaction between the effects of the circadian rhythms and those of a mechanical force, in vitro studies have the special advantage that different individual and biasing factors can be controlled to a higher degree. In our study, we applied a tensile strain of 20% which, according to Kanzaki et al., is rather strong, to induce tooth movement clinically [24]. A study applying a force of lower magnitude would certainly be of interest, as would be studies focusing on compressive strain. Furthermore, our measurements were limited to a period of 24 h. The reason for this were the findings of Hilbert et al. which suggested that without further synchronization the recognizability for the circadian rhythm of cells in vitro seems to weaken during 48 h [17].

### Clinical relevance

Currently there is still little information about the subject of precise timing of force strain and a periodicity pattern of orthodontic treatment. The question whether there is a time of the day when it is more effective to activate an appliance or to wear a removable appliance has not been investigated sufficiently. Miyoshi et al. demonstrated in rats that orthodontic tooth movement varied if the force was applied at different times of the day [32]. Applying identical forces, they found the amount of tooth movement to be reduced in animals that were kept in the dark for a longer period and, vice versa, prolonged exposure of the animals to light resulted in increased tooth movement as compared to animals kept in a normal day–night rhythm. In another animal study in rats, Xie et al. showed that the clock gene BMAL1 in PDL cells was involved in the process of bone remodeling during orthodontic tooth movement. Following orthodontic force application, BMAL1 expression in the periodontal tissues was upregulated and this upregulation was dependent on extracellular signal-regulated kinase (ERK) and activator protein 1 (AP1). Localized injection of the BMAL1 inhibitor GSK4112 suppressed ERK/AP1/BMAL1 signaling and, therefore, osteoclastic activity on the compression side was reduced [58].

With our study we tried to expand the understanding of the circadian rhythm in periodontal cells and in the long term to contribute to knowledge regarding effective wear time in orthodontic treatments.

## Conclusion

It seems that mechanical stress affects the expression of clock genes of the circadian rhythm in periodontal ligament (PDL) cells, which are at the same time involved in various other important cell functions. Furthermore, it appears that the cell response to the mechanical stimulus varies in the different phases of the circadian rhythm. Thus, our results could open the way to more efficient timing for orthodontic treatment devices in the future, especially for those appliances used on an hourly basis.
